# Acute postoperative spinal epidural hematoma following unilateral biportal endoscopic

**DOI:** 10.3389/fsurg.2026.1849549

**Published:** 2026-07-01

**Authors:** Jia Liu, Yingying Shi, Yuehuan Zheng, Peng Cao, Bin Yan, Xiaoning Wang

**Affiliations:** 1 Department of Operating Room, Ruijin Hospital, Shanghai Jiaotong University School of Medicine, Shanghai, China; 2 Department of Orthopedics, Ruijin Hospital, Shanghai Jiaotong University School of Medicine, Shanghai, China; 3 Shanghai Key Laboratory for Prevention and Treatment of Bone and Joint Diseases with Integrated Chinese-Western Medicine, Shanghai Institute of Traumatology and Orthopedics, Ruijin Hospital, Shanghai Jiaotong University School of Medicine, Shanghai, China

**Keywords:** epidural hematoma, muscular hemorrhage, neuropathic pain, spinal stenosis, unilateral biportal endoscopy (UBE)

## Abstract

**Objective:**

To report a rare case of acute postoperative epidural hematoma (PEDH) following unilateral biportal endoscopic (UBE) unilateral laminotomy with bilateral decompression (ULBD) for L3–4 spinal stenosis, presumably caused by muscular hemorrhage, and to explore its potential etiology through a review of the literature.

**Methods:**

The clinical data, including pre- and postoperative Visual Analog Scale (VAS) and Japanese Orthopaedic Association (JOA) scores, were recorded at key timepoints: preoperatively, pre-surgical drain removal, post-surgical drain removal, and post-emergency debridement. A literature review was conducted to contextualize the findings.

**Results:**

A 56-year-old male presented with left lower limb pain (VAS 6), intermittent claudication (walking distance: 100 meters), and a JOA score of 10 preoperatively. After undergoing the UBE-ULBD surgery at the L3-4 level, the patient reported significant relief of lower limb neurological symptoms upon regaining consciousness from anesthesia. However, three minutes after drain removal on the 24th hour postoperatively, the patient experienced sudden severe left lower limb pain (VAS 10), which was refractory to routine analgesics. Administration of morphine provided only partial relief (VAS 7). Physical examination disclosed localized swelling at the surgical site, and subsequent MRI imaging confirmed the presence of hematoma signals within the operative channel. Emergency debridement revealed continuous bleeding from a small muscular artery within the operative tract, which had led to hematoma accumulation and subsequent compression of the dural sac and nerve roots. The bleeding artery was successfully ligated, and the hematoma was meticulously evacuated. Following debridement, the patient's pain improved significantly (VAS 3), and he was discharged without any sequelae.

**Conclusion:**

Spinal postoperative epidural hematomas have been reported in 0.1%–3% of cases, however, this represents the first documented instance following UBE-ULBD surgery. Hemorrhage from the muscular layer is identified as a potential etiology, underscoring the necessity for meticulous intraoperative hemostasis, particularly after restoration of baseline blood pressure prior to wound closure. Routine placement of a drainage tube is therefore recommended. Immediate surgical intervention, preferably within 24 h, is crucial for alleviating neurological compression, as emphasized by expert consensus, and significantly improves functional outcomes.

## Introduction

Unilateral biportal endoscopic spine surgery (UBE) has emerged as a minimally invasive alternative for treating lumbar spinal stenosis, offering advantages such as reduced tissue trauma and faster recovery (1, 2). While complications like dural tears and infections are well-documented (3), acute epidural hematoma following UBE remains unreported. This case report describes a rare complication of muscular hemorrhage leading to acute epidural hematoma after UBE-ULBD and discusses preventive strategies based on existing literature.

## Case presentation

### Pro-operative exanimation

**Figure 1:**
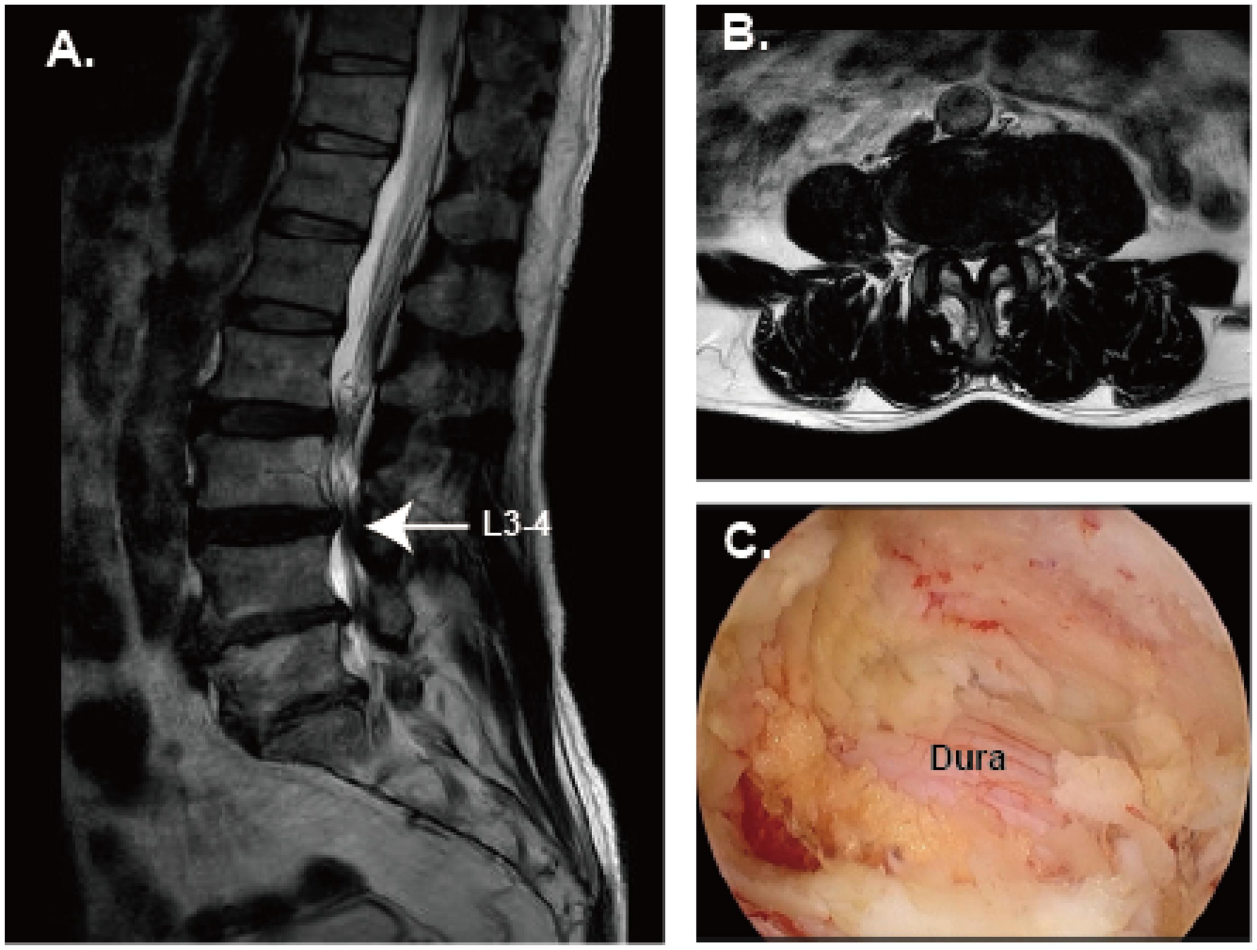
Preoperative MRI and intraoperative UBE images of the patient. Preoperative MRI indicated lumbar spinal canal stenosis at the L3-4 level **(A,B)**. Intraoperative visualization UBE confirmed adequate decompression of the thecal sac **(C)**.

A 56-year-old male presented with progressive left lower limb radicular pain (VAS 6) and intermittent claudication (limited to 100 meters) for two years. The patient underwent conservative treatment for more than three months, but the symptoms did not improve and progressively worsened. Physical exanimation indicated as Preoperative MRI confirmed L3-4 spinal stenosis without lumbar spondylolisthesis or lumbar instability, as depicted in Figures 1A,B.

### Surgical procedure

The patient decided to undergo UBE-ULBD to relieve the symptoms of lumbar spinal stenosis. The procedure was conducted with the patient in the prone position under general anesthesia and the preoperative level confirmation was performed using fluoroscopic guidance with a C-arm imaging system. For UBE-ULBD, two portals were utilized to establish the working space, each measuring approximately 1 cm in size. One served as the viewing portal, while the other functioned as the working portal. A continuous irrigation system was used and meticulously controlled to maintain an irrigation pressure at approximately 30–50 mmHg. A radiofrequency catheter was used to debride the soft tissue overlying the left side of L3-4 interlaminar space and an abrasive drill and Kerrison punch were utilized to perform a laminotomy. The partial laminectomy was subsequently conducted to adequately expose the ligamentum flavum. After flavectomy, the dura mater and bilateral nerve roots was exposure (Figure 1C). Subsequently, a nerve probe hook was used to meticulously remove the herniated intervertebral disc, and the intradural radiofrequency blade tip to ensure the absence of any clear active bleeding surrounding dura mater and nerve roots. Intraoperatively, hemostasis was achieved under controlled hypotension (mean arterial pressure: 70 mmHg), and a routine water-tight (saline irrigation) test was conducted to avoid undetected bleeding points. Finally, a closed suction drain was placed.

### Post-surgical epidural hematoma and emergency debridement

**Figure 2:**
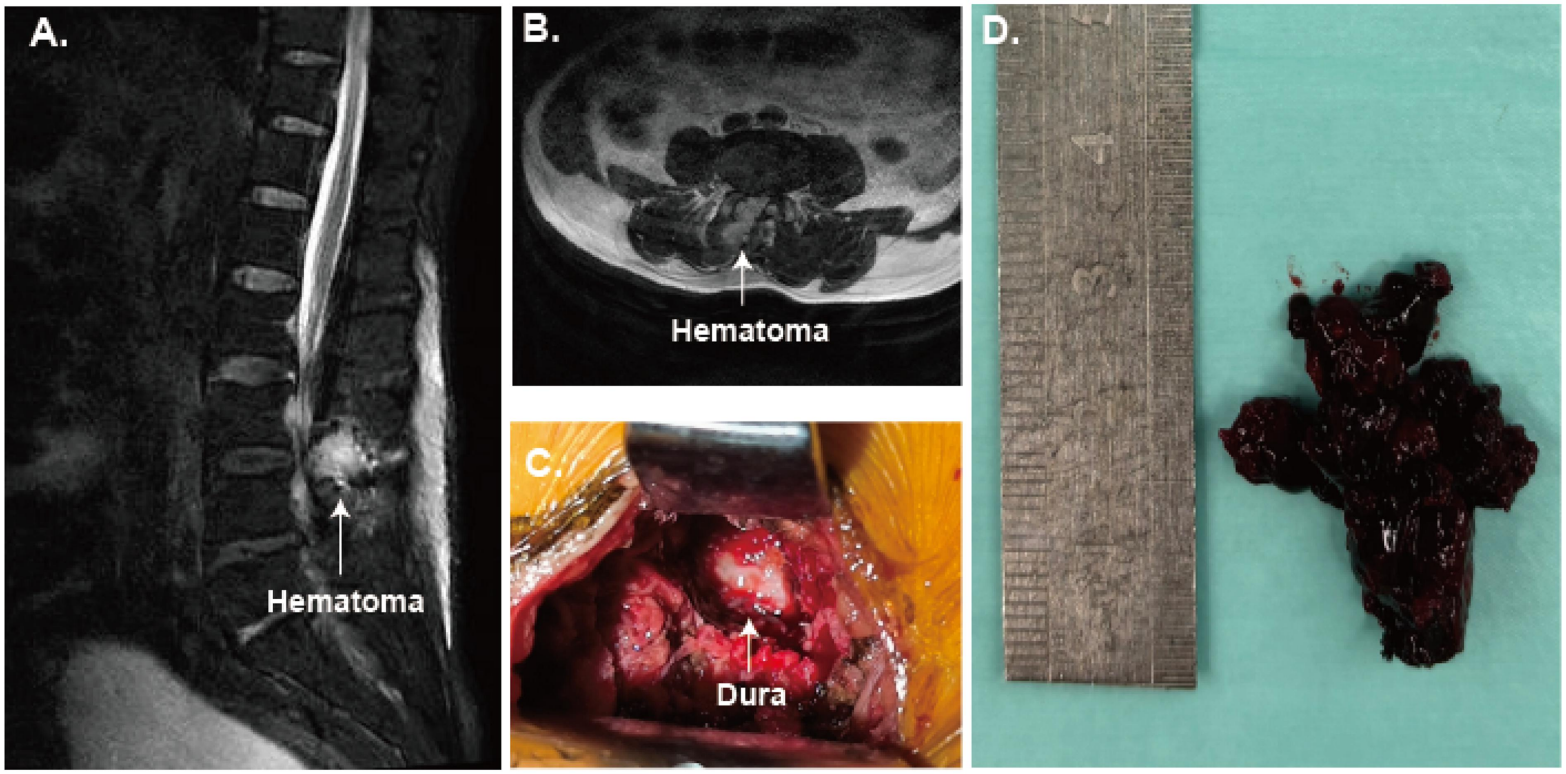
Postoperative hematoma imaging following UBE procedure. Postoperative MRI revealed epidural hematoma causing compression of the thecal sac **(A,B)**. Upon surgical re-exploration, an epidural hematoma compressing the dural surface was identified **(C)** Photograph of the evacuated hematoma **(D)**.

Postoperatively, the patient reported immediate alleviation of symptoms of sciatica. Approximately 25 mL of pale red and bloody fluid was drained from the surgical site, postoperatively. The drainage tube showed no obvious obstruction or dislodgement off the incision. At 24 h postoperatively, the surgical drain was removed after releasing the negative pressure suction. Within three minutes, the patient developed severe pain in the left lower limb (rated as 10 on the VAS), which was unresponsive to nonsteroidal anti-inflammatory drugs (NSAIDs). Administration of morphine reduced the pain to a VAS score of 7. Physical examination revealed localized swelling along the operative tract. An emergency MRI scan demonstrated a 4.5 cm × 2.0 cm hematoma within the operative channel (with the measured diameter of the actual blood clot was around 3 × 1.5 cm), resulting in compression of the dural sac and the L4 nerve root (Figures 2A,B).

The patient underwent emergent surgical exploration (Figure 2C), which identified active bleeding from a small muscular artery in the operative tract. The vessel was ligated, and the hematoma was evacuated. The size of the hematoma was around 3*1.5 cm, as depicted in Figure 2D. Postoperatively, the left lower limb pain decreased to VAS 3, and the patient regained full neurological function.

## Discussion

Symptomatic PEDH represent a rare but clinically significant complication, with profound postoperative implications for patients' functional outcomes, and most of the cases were from minimally invasive lumbar laminectomies (1). Nevertheless, epidural hematoma following spinal surgery does not necessarily induce symptoms in patients. Modi et al. reported that among 89 patients following microscopic lumbar decompression, postoperative MRI revealed epidural hematomas in 13 cases, yet only 2 exhibited neurological symptoms (2). Moreover, for patients with epidural hematoma accompanied by neurological symptoms, emergency elimination of the hematoma does not adversely affect long-term neurological recovery outcomes (2, 3). However, early detection of neurological deficits, immediate MRI, and timely surgery was needed to minimize postoperative neurological sequelae, and PEDH should not be ignored (4).

With the development of UBE technology, some scholars suggested that UBE may lead to an increased incidence of PEDH based on comparative study (5). The reported incidence of PEHD following UBE was around 0.27%–1.1% according to meta-analysis (6, 7). Although most cases are asymptomatic, symptomatic PEDH may result in acute neurological deterioration and irreversible deficits if not promptly treated (8). UBE-specific factors such as continuous irrigation and limited visualization of venous bleeding may contribute to delayed hematoma formation, potentially masking intraoperative bleeding sources (9). Therefore, despite its low incidence, PEDH after UBE should be considered a critical complication requiring early recognition and timely intervention.

However, the study about the reason of PEDH following UBE was rare. In this case, the patient had no abnormal coagulation function before the surgery and no history of taking anticoagulant drugs. During the UBE procedure, no obvious active bleeding was observed, and no significant bleeding was found in the postoperative drainage either. During the second surgical exploration, it was found that the bleeding from small muscular arteries. We hypothesized that this artery of the muscle or fascia was in a closed state when the drainage tube was compressed. However, when the drainage tube was removed, the small artery reopened and caused hematoma formation within the narrow space of the UBE, which subsequently led to neurological symptoms. Therefore, based on this particular case, in addition to hemostasis around the dura and nerve roots after UBE surgery, adequate hemostasis of the muscles and fascia is also essential to reduce the probability of PEDH.

Based on this case, several preventive strategies for acute postoperative hematoma following UBE can be proposed. First, meticulous hemostasis should be performed after restoration of normotension, as intraoperative hypotension may mask bleeding sources. Blood pressure has been shown to affect visualization of bleeding during UBE, and inadequate hemostasis under low pressure may lead to postoperative rebleeding (10). Therefore, it is recommended to elevate blood pressure to normal levels and re-assess the surgical field without irrigation before closure. Second, appropriate drainage is important for reducing hematoma risk. A prospective randomized study indicated that the number and placement of drain tubes may influence postoperative spinal epidural hematoma formation after UBE surgery (11). In general, drainage for 24–48 h is recommended, with adjustments based on intraoperative bleeding and patient condition. Finally, close postoperative neurological monitoring is essential, especially after drain removal. Delayed symptomatic hematoma, including remote hematoma, has been reported after UBE and may cause neurological deterioration (12). Early detection and prompt imaging are critical.

## Conclusion

This report presents the inaugural case of an acute epidural hematoma secondary to muscular hemorrhage following UBE-ULBD surgery. It highlights the imperative for surgeons to prioritize meticulous hemostasis, vigilant postoperative monitoring, and prompt intervention in the event of acute neurological deficits. Further studies are warranted to establish standardized protocols for the prevention and management of this rare complication.

## Data Availability

The raw data supporting the conclusions of this article will be made available by the authors, without undue reservation.
